# Physical Performance Is Associated with Working Memory in Older People with Mild to Severe Cognitive Impairment

**DOI:** 10.1155/2014/762986

**Published:** 2014-03-16

**Authors:** K. M. Volkers, E. J. A. Scherder

**Affiliations:** ^1^Department of Clinical Neuropsychology, VU University, Van der Boechorststraat 1, 1081 BT Amsterdam, The Netherlands; ^2^Center for Human Movement Sciences, University of Groningen, Antonius Deusinglaan 1, 9713 AV Groningen, The Netherlands

## Abstract

*Background*. Physical performances and cognition are positively related in cognitively healthy people. The aim of this study was to examine whether physical performances are related to specific cognitive functioning in older people with mild to severe cognitive impairment. *Methods*. This cross-sectional study included 134 people with a mild to severe cognitive impairment (mean age 82 years). Multiple linear regression was performed, after controlling for covariates and the level of global cognition, with the performances on mobility, strength, aerobic fitness, and balance as predictors and working memory and episodic memory as dependent variables. *Results*. The full models explain 49–57% of the variance in working memory and 40–43% of episodic memory. Strength, aerobic fitness, and balance are significantly associated with working memory, explaining 3–7% of its variance, irrespective of the severity of the cognitive impairment. Physical performance is not related to episodic memory in older people with mild to severe cognitive impairment. *Conclusions*. Physical performance is associated with working memory in older people with cognitive impairment. Future studies should investigate whether physical exercise for increased physical performance can improve cognitive functioning. This trial is registered with ClinicalTrials.gov NTR1482.

## 1. Introduction

In healthy older people a high level of physical activity coincides with a high level of cognitive performance, such as speed of information processing, attention [1], and executive functions (EF) [2]. The results of those studies are in line with the finding that a high level of physical activity during life might decline the risk of dementia [3]. Since physical activity also increases* physical performance*, such as muscle strength, gait speed, functional mobility, and balance [4], it is not surprising that there is a positive relationship between physical performance and cognition in healthy older people [5]. More specifically, older people with better physical performance levels, for example, mobility [6], balance [7], strength [6, 8], and aerobic fitness [9], have better cognitive functions, such as cognitive flexibility or global cognition. Moreover, similar to physical activity, better physical performance, such as balance [7] and strength [8, 10], also decreases the risk of dementia [11].

The studies mentioned above suggest a close relationship between physical performance and cognitive functioning in cognitive healthy older people. In older adults with amnestic mild cognitive impairment (aMCI) [12] or mild dementia [13], this relationship is further strengthened. In people with aMCI, gait speed and the performance on the Timed Up and Go (TUG) were both associated with EF [12], which are higher cognitive functions, such as working memory, supported by the prefrontal cortex (PFC) [14]. It is even suggested that particularly EF, as opposed to global cognition or memory, is important for mobility performances, such as balance, gait [15], and the ability to perform the activities of daily life (ADL) [16]. This suggestion was supported by a positive relationship between gait and EF in a combined group of cognitive healthy young elderly and elderly with and without mild dementia [17].

Not only is gait affected in an early stage of dementia [13, 18–20], but also there is increasing evidence for a decline in lower-extremity functioning, for example, walking speed [19], balance [18, 20], fine and complex motor functioning [21], aerobic fitness [22], and limb coordination [20], already in an early stage of dementia. When people have dementia in a relatively early stage, balance stays an independent predictor of the progression in (further) global cognitive decline [23]. The studies above, which included people with dementia, show only a relation between physical performance and the stage or progression of the dementia, not with specific cognitive functions. In addition, these studies often include only specific types of dementia or only people in, for example, a mild stage of dementia, and often did not control for the level of depression, even though this can influence the level of cognition [24].

The goal of the present study was to examine if physical performance (strength, balance, mobility, and aerobic fitness) is related to specific cognitive functions in people with mild to severe cognitive impairment. If this appears to be the case, therapeutic interventions specifically aimed at maintaining or improving one or more physical performances might be useful to slow down a decline or even to improve cognitive functioning in cognitively impaired older people.

## 2. Methods

The present cross-sectional study includes baseline data of a longitudinal randomized controlled trial (RCT) examining the effect of physical activity on, among others, physical performance and cognition (for details, see [25]). Participants were recruited via medical staff of aged care facilities. Firstly, the medical staff was informed about the goal and procedure of the RCT. Secondly, possible participants were selected within subunits of the institutions. Thirdly, an information letter with informed consent was sent to the legal representatives of the selected participants. Fourthly, once written consent was received, participants were tested for inclusion and exclusion criteria.

### 2.1. Participants

**Figure 1:**
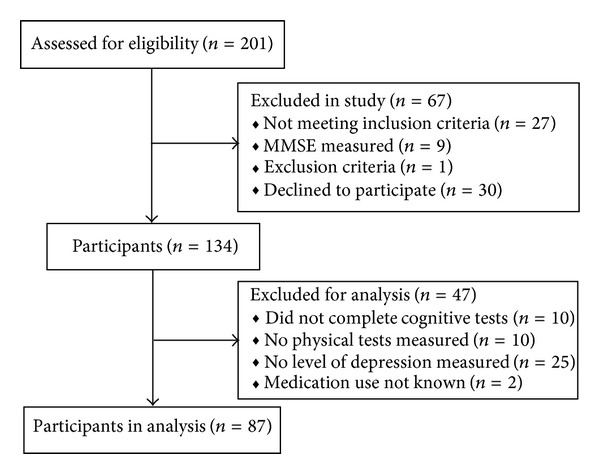
Flowchart of participants.

One hundred and thirty-four participants (96 women), 82.2 ± 7.4 years old, with cognitive impairment participated in this study. However, due to missing values, 47 participants could not be analysed (see Figure 1). The severity of the cognitive impairment was determined by the Mini-Mental State Examination (MMSE), a test to measure global cognitive functioning, that is, orientation in time and place, word recall, attention and calculation, language abilities, and visuospatial ability (scores range from 0 to 30) [26]. Eligibility criteria for study participation were the presence of cognitive impairment (MMSE < 25) and being ambulatory with or without walking aid (walker or cane). Exclusion criteria were the presence of personality disorders, cerebral traumata, hydrocephalus, neoplasm, disturbances of consciousness, and focal brain disorders. Characteristics of participants are shown in Table 1.

### 2.2. Level of Depression

The level of depression was based on the summed standardized scores of Geriatric Depression Scale (GDS) and Symptoms Checklist 90 (SCL-90) (Cronbach's Alpha is 0.91).

*GDS. *The Dutch version [27] of the GDS is a 30-item questionnaire used to measure general mood [28]. The GDS is a reliable and valid self-rating depression screening scale for elderly populations [29]. The GDS questions are answered by “yes” or “no” depending on which response is most appropriate at the time of measurement, with 0 or 1 point for each answer. Higher scores indicate a higher level of depression with a maximum score of 30.

*SCL-90.* One subscale from the Dutch version [30] of the SCL-90, a 90-item self-report symptom inventory designed to reflect patterns of current psychological symptoms, was used to measure depression [31, 32]. The depression subscale includes 15 items. Each item is rated on a 5-point likert scale, from 1 (not at all) to 5 (extremely). A higher score indicates more symptoms of depression with a maximum score of 75.

### 2.3. Education

The highest education level was determined on a seven-point scale with 1 = less than elementary school to 7 = university and technical college [33].

### 2.4. Comorbid Conditions

Comorbid conditions (see Table 1) were extracted from the medical status and categorized based on the Dutch translation of the Long-Term Care Facility Resident Assessment Instrument (RAI), section I. This section (disease diagnoses) includes the following categories: (1) endocrine/metabolic/nutritional, (2) heart/circulation, (3) musculoskeletal, (4) neurological, (5) sensory, (6) psychiatric/mood, (7) pulmonary, and (8) others. The total sum of 8 categories was used as a comorbidity score.

### 2.5. Medication Use

Medication use (see Table 1) is coded according to the Dutch Pharmacotherapeutic Compass and is ranged by the following groups: (1) antipsychotics, (2) antidepressants, (3) pychotropics (central nervous system (CNS)), (4) neurological (CNS), (5) anaesthetics and muscle relaxing, (6) blood, (7) cardiovascular, (8) gastrointestinal tract, (9) respiratory tract, (10) kidneys and urinary tract, (11) genital tract, (12) dermatology, (13) otolaryngology, (14) ophthalmologic, (15) infectious diseases, (16) hormones and bone metabolism, (17) analgesics, antirheumatic drugs and gout agents, (18) vitamins and minerals, (19) malignancies, (20) infectious diseases, (21) various preparations, (22) dentistry, and (23) opioids. The total of all 23 categories was used as medication score.

### 2.6. Informed Consent

The Medical Ethical Committee of VU university medical center approved the longitudinal study. Before the baseline measurement, participants or their caregivers provided written informed consent for the longitudinal study.

## 3. Outcome Variables

To assess physical performance and cognitive functioning, the following tests were administered.

### 3.1. Assessment of Physical Performance

#### 3.1.1. Mobility

The mobility performance was computed by three physical tests, that is, the Ten Meter Timed Walk, Figure of Eight, and the TUG (Cronbach's alpha = 0.86). For final mobility, the performance was multiplied by −1, where higher scores indicate better mobility.

*Ten Meter Timed Walk.* Participants are requested to walk 10 meters at their own regular pace between 4 small traffic cones, which are placed in the corners of a 10 by 1 meter rectangle [34]. The time to walk 10 meters is measured by hand with a stopwatch to the nearest of 1/10 of a second.

*Figure of Eight.* The Figure of Eight is an applicable and reliable dynamic functional balance measure of mobility for people with various degrees of physical disability [35] and geriatric patients [36]. The Figure of Eight test requires continuous turning with an emphasis on accuracy (avoid oversteps), speed (timed task), and switching of motor patterns during the crossover from the clockwise to the counterclockwise loop. Participants are timed while walking in a figure-8 trajectory. The figure-8 trajectory is marked with white paint on a dark green rubber carpet, each loop having an outer diameter of 165 centimetres (cm) and a step width of 15 cm. The time to walk two complete eight figures is measured with a stopwatch. The onset time is based on the first detectable movement of the participant following a “Go!” command from the observer. Any step taken outside the white line is noted. The fastest attempt of two trials is recorded together with the corresponding oversteps.

*TUG.* The TUG is a reliable and valid test for quantifying functional mobility that may also be useful in following clinical change over time [37]. To complete the TUG, participants are requested to rise from a standard chair (48 cm height, horizontal seat with armrests), walk 3 meters, turn around, and return to a fully seated position in the chair again [38]. Each participant has two trials and the average time in seconds is the outcome of the TUG.

#### 3.1.2. Strength

*Sit To Stand (STS).* The STS is normally a reliable and valid indicator of lower body strength in adults over 60 years [39]. However, in this study, participants are allowed to use upper limbs to rise from the chair to test their rising performance that is closest to the clinical setting and to reduce a floor effect; a high percentage of older dependent elderly cannot rise from a chair with the arms crossed in front of the chest [40]. Participants are instructed to stand up and sit down in a standard chair as many times as possible within 30 seconds. The STS score is formed by the total number of performances with a sit-stand-sit performance counting as 1. Ending in a standing position is counted by a 0.5 point.

#### 3.1.3. Aerobic Fitness

*Six-Minute Walk Test (6 MWT).* The 6 MWT can be used reliably in the assessment of functional endurance ambulation in persons with acquired brain injury [41]. During the performance of the 6 MWT, participants are instructed to cover as much distance as possible during 6 minutes with the opportunity to stop and rest if necessary [42]. Participants have to walk around a premeasured, unobstructed 10 by 1 meter rectangular circuit having semicircular ends with 0.5 meter radii marked out with plastic cones to prevent participants having to walk at sharp angles. One full round covers 26.3 meters walking. The total walking distance by each participant will be measured to the nearest meter.

#### 3.1.4. Balance

*Frailty and Injuries: Cooperative Studies of Intervention Techniques (FICSIT-4).* The FICSIT-4 is a test to measure static balance [43]. The participants have to maintain balance in 4 positions with increasing difficulty. Each position is demonstrated first and support is offered while participants position their feet. When participants are ready, the support will be released and timing begins. The timing stops when participants move their feet or grasp the researcher for support, or when 10 seconds have elapsed. Only when one position is performed for 10 seconds, the next, more difficult position is performed. The first position is with the feet together in parallel (side-by-side) position. Second is the semitandem position: the heel of one foot is placed to the side of the first toe of the other foot. The participant can choose which foot to place forward. Third is a tandem position: the heel of one foot directly in front of the toes of the other foot. The final position is standing on one leg. The total summed seconds of the performed positions are the outcome score.

### 3.2. Assessment of Cognitive Functioning

Besides the MMSE, 13 neuropsychological tests were administered, but 6 tests, that is, Digit Span forward, Visual Memory Span forward, Rule Shift Cards, Key Search, Picture Completion, and the Stroop test (for details, see [25]), were not analyzed in this study, because these tests could not be included in a specific cognitive domain. Four tests, that is, the Digit Span backward and Visual Memory Span backward, Category Fluency tests, and the Digit Symbol Substitution Test (Cronbach's alpha = 0.82), could be included into one domain, that is, working memory (for processing information), one of the EF. Furthermore, 3 tests, that is, the Eight Words test and Face Recognition and Picture Recognition (Cronbach's alpha = 0.75), could be combined to compose an episodic memory domain (for learning new information).

### 3.3. Working Memory

*Digit Span Backward.* The Digit Span is a subtest from the Wechsler Memory Scale-Revised (WMS-R) [44]. In the Digit Span backward, increasingly long sequences of random numbers are orally presented at a rate of one digit per second to the participants, who have to repeat the sequence in reverse order immediately after oral representation. This condition ends when a participant fails to recall at least two strings of the same length or repeats an eight-digit sequence correctly. The minimal score for this conditions is 0 and the best score is 21.

*Visual Memory Span Backward.* The Visual Memory Span is a subtest of the WMS-R [44]. The Visual Memory Span backward stimuli consist of squares printed on a two-dimensional card and requires the participant to repeat a number of tapping sequences in reverse order, similar to the Digit Span backward. This test is used as a measure of visual working memory [44]. Scores range from 0 (worst) to 12 (best).

*Category Fluency Test.* The Category Fluency test is a verbal fluency test which can be used to evaluate working memory [45]. The participant is asked to name as many examples of a given category as possible, within 1 minute. This study uses the category “animals” and “professions” [46]. The outcome measure is the total number of animals and professions produced.

*Digit Symbol Substitution Test (DSST).* The DSST is a subtest of the WAIS-Revised [44]. Test scores correlate with general intelligence, cognitive impairment, chronological age, and activation in the frontal regions [47–49]. Participants are presented with a rectangular grid of numbers. For each of these numbers, participants are instructed to substitute the appropriate symbol according to a code that appears at the top of the page. The DSST score is recorded as the number of correct symbols drawn in 2 minutes.

### 3.4. Episodic Memory

*Eight Words Test.* The Eight Words test is a list-learning test for people with memory problems [50]. In this test, the examiner reads out eight words in a row, which is repeated five times. Every time the participant is asked to recall as many words as possible. The first outcome measure is the total number of correctly recalled words after the five trials (*immediate recall score*, maximal score = 40). After an interval of approximately 15 minutes, the participant is asked to recall as many words as possible (*delayed recall score*, maximal score = 8). Subsequently, the examiner reads aloud 16 words among which 8 words presented before and 8 new words. The participant is asked to recognize the words from the list presented before (*recognition score*, maximal score = 16).

*Face Recognition.* Face Recognition is a subtest from the Rivermead Behavioral Memory Test (RBMT) [51] and measures visual, nonverbal long-term memory. Two versions (C+D) are combined to prevent a ceiling effect. In this test, the participant is shown 10 cards with faces one at a time for 5 seconds. After a short interval of approximately 2 minutes, the participant is shown 20 cards, including 10 shown before and 10 cards with new faces. The participant has to recognise whether the card was shown before or not. The outcome measure is the number of faces correctly recognized minus the number of faces incorrectly recognized. The worst score is −20 and the best score is +20.

*Picture Recognition.* Picture Recognition is also a subtest from the RBMT [51], which measures visual, verbal long-term memory. Two versions (C+D) are combined to prevent a ceiling effect. The participant is shown each of the 20 cards with drawings of objects for 5 seconds. With each card, the participant is requested to name the object on the card. After a short interval of approximately 2 minutes, the participant is shown 40 cards, including 20 shown before and 20 cards with new objects. The participant has to recognise whether the card was shown before or not. The outcome measure is the number of objects correctly recognized minus the objects that were incorrectly recognized. The lowest score is −40 and the maximal score is +40.

## 4. Data Analysis

The data was analyzed using* Statistical Package for the Social Sciences *(*SPSS*) version 16.0 (SPSS, Inc., Chicago, IL). For data reduction, scores on neuropsychological tests and physical performances were converted into standardized *z*-scores to receive equal weighting towards a combined domain. With principal component analysis, eigenvalues >1, at least a good internal consistency of the domain (Cronbach's alpha at least 0.70), 2 cognitive domains, and 1 physical performance domain could be developed by summing up the *z*-scores. The 3 other physical performances were based on 1 test. Hierarchical multiple regression analysis involved four steps. We tested the hypothesis that a physical performance (mobility, balance, strength, or aerobic fitness) would be a significant predictor of cognitive functioning (working memory or episodic memory) (Step 3) after controlling for age, education, depression, comorbidities, medication use (Step 1), and cognitive impairment (MMSE) (Step 2). The significance of the increment in the squared multiple correlation was tested when the physical performance was entered after the control variables. Furthermore, to analyze whether the physical performance as a predictor was different for people in different stages of cognitive impairment, we added the interaction (MMSE × physical performance) to the model (Step 4). A two-sided *P* value < 0.05 was considered statistically significant.

## 5. Results

The results of the hierarchical multiple regression analysis with working memory and episodic memory as dependent variables, controlling for age, education, depression, comorbidity, medication (Step 1), MMSE (Step 2), the physical performance (Step 3), and interaction between physical performance and MMSE (Step 4) as predictors are shown in Table 2.

### 5.1. Working Memory

*Balance*,* strength,* and* aerobic fitness* (Step 3) are all significantly associated with working memory (*P* < 0.05) after controlling for covariates and the level of global cognition. Each performance explains 3% to 7% of the total variance of working memory, irrespective of the level of cognitive impairment (Step 4 not significant).

### 5.2. Episodic Memory

*Mobility*,* balance*,* strength,* and* aerobic fitness* (Step 3) are not significantly related to episodic memory (*P* ≥ 0.05) after controlling for covariates and the level of global cognition in older people with all levels of cognitive impairment (Step 4 not significant).

## 6. Discussion

Although physical performances, such as strength and mobility, are assumed to be related to cognition, for example, global cognition measured with 19 different neuropsychological tests [10] and EF [6, 12], our findings show that in people with mild to severe cognitive impairment, this is also true for working memory, but not for episodic memory. More specifically, the results indicate that the performance in* balance*,* strength,* and* aerobic fitness* is positively related to the performance in working memory, irrespective of the level of cognitive impairment.

### 6.1. Working Memory

*Strength*,* balance,* and* aerobic fitness* are significantly associated with working memory, an aspect of EF [14], in people with a mild to severe cognitive impairment. This association is independent of the number of comorbidities, age, level of depression, education level, and medication use. That* strength* (also measured with STS) is related to working memory/attention, measured by the Digit Span forward and backward, was also observed in older cognitively healthy women [52]. In contrast, in a combined group of cognitively healthy older men and women, knee extension strength was not related to working memory [6]. However, in that study working memory was assessed by only one neuropsychological test, that is, the Digit Span backward. In addition, knee extension strength was related to a Lexical Fluency test [6]. The latter test measures cognitive flexibility, which is in the current study included in the working memory domain by two Category Fluency tests [53]. In the current study, the Digit Span backward and two Category Fluency tests were only three out of five neuropsychological tests of a strong domain “working memory” (Cronbach's alpha = 0.85). Overall, all of the measured working memory and cognitive flexibility tests mentioned above appeal to EF [25], and therefore* strength* seems to be related to EF and not only working memory.

In the present study,* mobility* was not significantly associated with working memory in people with a cognitive impairment. A possible reason why mobility is not related to working memory, while other physical measures are, is that mobility was performed at “regular pace” instead of “as fast as possible”, which probably causes a smaller difference in performance between participants. In cognitively healthy older people, the 4-meter timed walk test, which was also performed at usual pace, was also not related to the Digit Span test (score of Digit Span backward minus the score on the Digit Span forward) in older women [54] nor was the mobility performance (measured with POMA) related to the Digit Span backward in older men and women [6]. In contrast, the mobility performance of the latter study was associated with fluency, a cognitive performance that we included in working memory. However, their mobility performances (measured with the POMA) included not only gait and mobility but also balance. Possibly balance caused the significant relation, since* balance* is also significantly associated with working memory in the present study.* Balance* is dependent on the functioning of the frontocerebellar and frontostriatal connections [13], connections between, respectively, the cerebellum and the striatum and the frontal cortex, for example, dorsolateral PFC (DLPFC) [13]. Since the DLPFC is also involved in working memory [55], it is not surprising that in people with a cognitive impairment,* balance* is significantly related to working memory, because both performances appeal to the same neural circuits.

*Aerobic fitness* (measured with 6 MWT) is significantly associated with working memory in cognitively impaired older people. This was not observed in cognitively healthy older women [52]. However, the latter study measured a small number of participants (*n* = 41) and had a combined EF domain of working memory with attention. This combined domain was measured with the Digit Span backward and with the Digit Span forward, which is different from the current study. A mechanism underlying the present finding might be that* aerobic fitness* is associated with white matter volume, even after controlling for age, gender, dementia severity, physical activity, and physical frailty [22]. White matter volume is positively related to working memory [56]. Indeed, executive control processes, such as working memory, show the largest benefits of improved fitness in older people [56]. Clinically, working memory is essential for storing information, and therefore it is crucial for long-term memory and learning [57, 58]. However, working memory is vulnerable during aging and dementia [59]. To reduce a decline in working memory, results of this study suggest that it is important to maintain good balance, strength, and aerobic fitness. Indeed, in older people with mild Alzheimer's disease, balance and coordination exercises seem to improve working memory in a pilot study [60].

### 6.2. Episodic Memory

*Mobility*,* balance*,* strength,* and* aerobic fitness* are not related to episodic memory in people with mild to severe cognitive impairment. These nonsignificant results are not very surprising, since motor performances are highly related to PFC-related cognitive functions, for example, attention, EF, and working memory, and less with hippocampal cognitive functions, for example, episodic memory. Therefore,* aerobic fitness* interventions show the highest effect sizes on cognitive functions in which the PFC plays an important role [56]. However, a higher effect size does not imply that aerobic fitness is only related to PFC-related cognitive functions and not with hippocampal related cognitive functions. Indeed, a comparable study in older people with MCI has suggested that aerobic fitness may be the most important physical performance, besides strength, balance, and mobility, that is related to the volume of the hippocampus [61]; this has been confirmed in another study in people with (very) mild AD [62]. Because hippocampal volume is positively related to episodic memory [63], these studies suggest that aerobic fitness and episodic memory are associated with people with MCI and (very) mild AD. However, participants of both studies were not only 8 years younger than participants of the current study (74 versus 82 years), but they had less cognitive impairment as well, including also people with subjective cognitive impairment, with a mean MMSE of 27 [61] or 26 [62]; in the current study participants with a MMSE above 24 were excluded. With increasing cognitive impairment, the hippocampus and PFC are both more affected [64]. However, to encode items for episodic memory, the anterior medial PFC is activated as well [65]. This suggests that, in people with increasing cognitive impairment, a high level of aerobic fitness, obtained by a high level of physical activity, has to improve the affected PFC first before an improvement in episodic memory can be observed. Therefore, we argue that the relationship between aerobic fitness and working memory (or EF) is stronger than the relationship between aerobic fitness and (episodic) memory in people with cognitive impairment. Indeed, in people with a decline in both working memory and episodic memory as is the case in obese older people [66], aerobic fitness was related to EF, but not to memory [67]. In cognitively healthy people with a well-functioning PFC, the relationship between aerobic fitness and episodic memory is more often observed [68–71]. As far as the authors know, there are no other comparable studies assessing the relation between specific physical performance and episodic memory in older people with objective mild to severe cognitive impairment.

### 6.3. Passivity

Physical performances can be increased by physical activities not only in cognitively healthy older people but also in people with a cognitive impairment [72]. Since physical performances are related to cognitive functioning, it is not surprising that cognitive functioning decreases faster in people with low levels of physical activity [73]. Regrettably, most elder community-dwelling people do not meet the recommended level of physical activity [74], which is at least 30 minutes of moderate intensity for 5 days per week in sessions of at least 10 minutes [75]. Cognitive functions decline even faster when people move into an institution, because of their low levels of physical activity [74]. Therefore, we need to consider the optimal timing and intensity of the physical activity, as well as the type of training, which should improve balance, strength, and aerobic fitness.

### 6.4. Limitation

A limitation of the present study is its cross-sectional design, implying that one can only report associations instead of a causal relationship. Longitudinal intervention studies are necessary to examine whether improvements in physical functioning also increase cognitive functioning, such as working memory.

Another potential limitation of our study is the composition of our study sample. Participants were recruited for a longitudinal RCT. Consequently, our sample may represent only participants who are willing to be randomised to an experimental or control group and are willing to attend multiple measurements. However, it was communicated that if participants were randomised into the control group, they were able to start the intervention at later moment, and that participants could refuse measurements at any time without reason.

## 7. Conclusion

In people with mild to severe cognitive impairment, the performances in balance, strength, and aerobic fitness are significantly associated with working memory, but not with episodic memory. Future studies should investigate whether physical exercise for increased physical performance can improve cognitive functioning. For the best physical exercise, we need to consider the optimal timing and intensity of the physical exercise, as well as the type of training, which should improve balance, strength, and aerobic fitness.

## Figures and Tables

**Table 1:** Demographics and characteristics of participants.

	Total participants (*n* = 134)		Participants in analysis (*n* = 87)		Participants not in analysis (*n* = 47)		Test statistics	
	mean	SD	mean	SD	mean	SD	*t*	df
Demographics and characteristics								
MMSE (0–30)	15.4	5.9	17.4	4.8	11.6	6.1	−5.66**	132
Age (years)	82.2	7.3	82.5	7.1	81.4	7.8	−0.84	132
Education (1–7)	3.4	1.5	3.4	1.4	3.4	1.7	0.12	127
Gender (% women)	71.6		75.9		63.8		2.18^*χ*^	1
GDS (0–30)	7.2	5.5	7.1	5.7	8.5	3.7	0.85	96
SCL-90 (0–75)	21.1	6.5	21.0	6.6	21.4	5.1	0.17	94
BMI (kg/m^2^)	26.9	4.4	27.2	4.3	26.3	4.5	−1.01	115
Medication use (0–26)	4.7	2.5	4.6	2.5	5.0	2.4	0.81	129

Comorbidities (% with disease)								
Endocrine/metab/nutr	24.2		26.4		20.0		0.67^*χ*^	1
Heart/circulation	65.2		66.7		62.2		0.26^*χ*^	1
Musculoskeletal	37.9		41.4		31.1		1.33^*χ*^	1
Neurological	97.0		96.6		97.8		0.15^*χ*^	1
Sensory	24.2		26.4		20.2		0.67^*χ*^	1
Psychiatric/mood	24.2		25.3		22.2		0.15^*χ*^	1
Pulmonary	9.8		9.2		11.1		0.12^*χ*^	1
Other	30.3		29.9		31.1		0.02^*χ*^	1

Notes: Test statistics show differences between participants in analysis and participants not in analysis; df: degrees of freedom; *t*: independent *t*-test; ^*χ*^ *χ* ^2^ test; *P value < 0.05; **P value <0.01.

BMI: Body Mass Index; GDS: Geriatric Depression Scale; metab: metabolic; MMSE: Mini-Mental State Examination; nutr: nutritional; SCL-90: Symptoms Checklist 90.

**Table 2:** Results of multiple regression analysis with physical performances as predictors (Steps 3 and 4) of working memory and episodic memory after controlling for age, education, level of depression, number of comorbidities, medication, and MMSE (Steps 1 and 2).

Dependent variable	Steps of analysis		Predictor	*β*	*t*	Cum *R* ^2^	Incr *R* ^2^
Working memory (*n* = 86)							
	Step 1		Age	−0.23	2.12*		
			Education	0.26	2.50*		
			Depression	−0.22	2.13*		
			Comorbidity	−0.04	0.29		
			Medications	−0.13	1.10	0.17	0.17*
	Step 2		MMSE	0.61	7.01**	0.49	0.32**
		Step 3	Mobility	0.08	0.77	0.49	0.00
		Step 4	MMSE ∗ mobility	0.18	0.78	0.50	0.00
		Step 3	Balance	0.20	2.02*	0.51	0.03*
		Step 4	MMSE ∗ balance	0.15	0.39	0.52	0.00
		Step 3	Strength	0.31	3.43**	0.56	0.07**
		Step 4	MMSE ∗ strength	0. 81	1.79	0.57	0.01
		Step 3	Aerobic fitness	0.21	2.02*	0.51	0.03*
		Step 4	MMSE ∗ aerobic fitness	0.46	1.22	0.52	0.01

Episodic memory (*n* = 87)							
	Step 1		Age	−0.29	2.60*		
			Education	0.19	1.82		
			Depression	0.07	0.62		
			Comorbidity	0.05	0.36		
			Medications	0.06	0.46	0.12	0.12
	Step 2		MMSE	0.58	6.20**	0.40	0.29**
		Step 3	Mobility	−0.05	0.45	0.40	0.00
		Step 4	MMSE ∗ mobility	−0.14	0.54	0.41	0.00
		Step 3	Balance	0.14	1.31	0.42	0.01
		Step 4	MMSE ∗ balance	−0.54	1.31	0.43	0.01
		Step 3	Strength	−0.07	0.72	0.41	0.00
		Step 4	MMSE ∗ strength	−0.40	0.76	0.41	0.00
		Step 3	Aerobic fitness	−0.00	0.02	0.40	0.00
		Step 4	MMSE ∗ aerobic fitness	−0.34	0.81	0.41	0.01

Notes: *β*: standardized beta coefficient; Cum: cumulative; Incr: increase; MMSE: Mini-Mental State Examination; *t*: *t* statistic. *P value <0.05; **P value <0.01.
